# The influence of artificial intelligence on the work of the medical physicist in radiotherapy practice: a short review

**DOI:** 10.1259/bjro.20230003

**Published:** 2023-10-19

**Authors:** Emmanuel Fiagbedzi, Francis Hasford, Samuel Nii Tagoe

**Affiliations:** 1 Department of Medical Imaging Technology and Sonography, University of Cape Coast, College of Health and Allied Sciences, Cape Coast, Ghana; 2 Department of Medical Physics, Accra-Ghana, University of Ghana, Accra, Ghana

## Abstract

There have been many applications and influences of Artificial intelligence (AI) in many sectors and its professionals, that of radiotherapy and the medical physicist is no different. AI and technological advances have necessitated changing roles of medical physicists due to the development of modernized technology with image-guided accessories for the radiotherapy treatment of cancer patients. Given the changing role of medical physicists in ensuring patient safety and optimal care, AI can reshape radiotherapy practice now and in some years to come. Medical physicists’ roles in radiotherapy practice have evolved to meet technology for the management of better patient care in the age of modern radiotherapy. This short review provides an insight into the influence of AI on the changing role of medical physicists in each specific chain of the workflow in radiotherapy in which they are involved.

## Introduction

**Figure 1.**
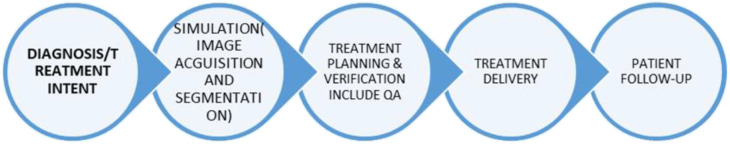
Traditional workflow in radiotherapy practice without AI. The workflow begins with the decision to treat the patient with radiation therapy, followed by a simulation appointment during which medical images are acquired for treatment planning. Subsequently, the patient-specific treatment plan is created, and then the plan is subjected to approval, review and QA measures prior to delivery of radiation to the patient. The patient then receives follow-up care.

Radiotherapy (RT) is one of the treatment modalities used to treat cancer. It can be used in the treatment of more than 50% of cancer patients, either as a single modality or in combination with surgery, chemotherapy. It has been estimated that around half of all cancer patients worldwide require radiation therapy. Due to the increasing incidence of cancer, the number of people getting treatment has also increased.^1^ A medical physicist (MP) is a health professional and specialist in radiation medicine who holds at least a master’s degree or equivalent in physics or engineering with requisite clinical training, whose functions in a radiation center cannot be underestimated.^2^ A medical physicist can provide a unique perspective on the various aspects of radiation oncology and medical imaging. In radiation oncology, he or she collaborates with the radiation oncologist and other members of the RT team to ensure that the treatment is complete and accurately delivered.^3,4^ In RT practice, medical physicists are engaged in both external beam RT and brachytherapy. Their major and typical roles include simulation, treatment planning, quality assurance, and treatment verification and delivery. Although Figure 1 represents the generalized workflow in a RT setting, the precise details may differ among different centers across the world. MPs in RT practice are also engaged in teaching, research, radiation protection and safety measures.^5,6^

## Artificial intelligence

Artificial intelligence (AI) has become more prevalent in the field of medicine and other fields. Its application in the field of RT has been discussed by many authors.^5,7–10^

**Figure 2.**
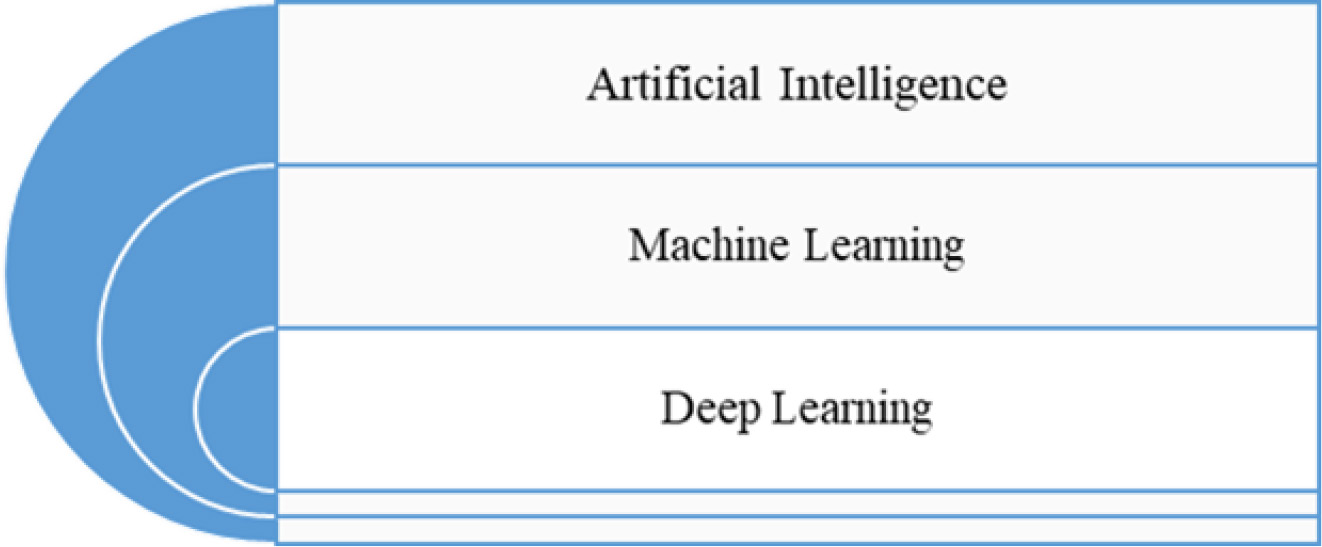
Relationship between artificial intelligence, machine learning and deep learning.

AI was first introduced in the United States in 1956 during the Summer Research Project at Dartmouth College. AI is a discipline focused on making intelligent machines. These machines can perform various tasks related to human intelligence.^11,12^ AI is a general term made up of the summation of two other terms namely machine learning and deep learning as shown in Figure 2.^13^

Machine learning is a type of AI that applies data-driven algorithms to copy human habits. Deep learning is another type of AI that uses deep neural networks to develop models.^6,14^ In this short review, the changing role of the medical physicist in the era of AI in RT practice has been presented.

### AI algorithms

**Figure 3.**
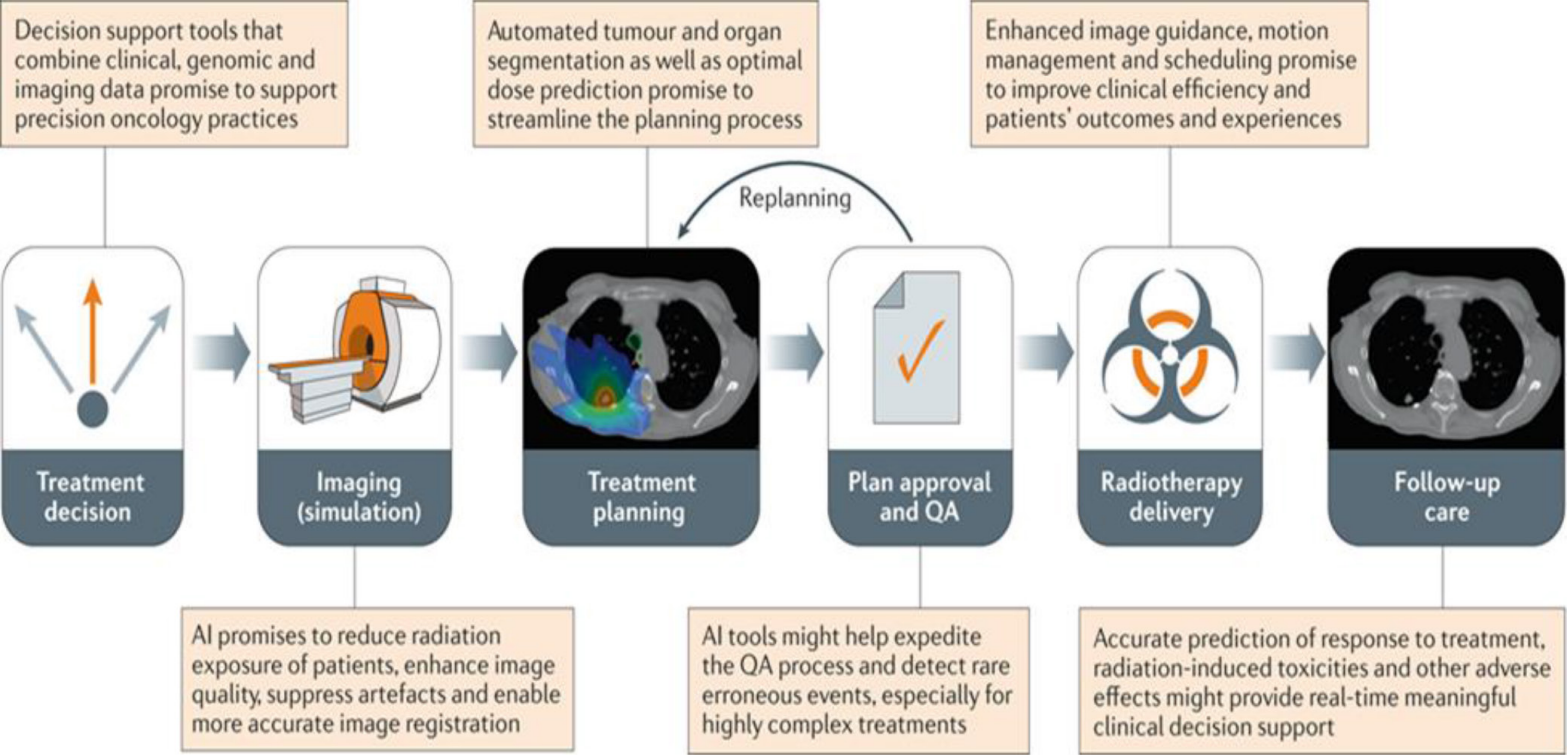
a summary of the influence of AI at each stage of the workflow involving medical physicists according to Huynh et al. AI has the potential to improve radiation therapy for patients with cancer by increasing efficiency, improving the quality of treatments, and providing additional clinical information and predictions of treatment response to assist and improve clinical decision-making ^8^.

AI algorithms are classified into four types: supervised learning, unsupervised learning, semi-supervised learning and reinforcement. The primary distinctions between these algorithms are in how they are trained and how they operate.(Figure 3)^15^

### Supervised learning

Supervised learning is a machine learning task of learning a function that maps an input to an output based on example of input–output pairs. This is the first and commonly used type of AI algorithms.^16,17^ A supervised learning algorithm learns the regression/classification model from a known set of input data and output. Then, a learning algorithm is used to train a model and predict how it will react to new data or a test data set. Examples of such learning algorithms include random forest, gradient boosting, neural networks (NNs), decision trees etc.^15,18,19^ In RT practice, whenever a new IMRT plan is prepared, this can be subjected to a trained prediction model to provide a pass/fail analysis. Valdes et al,^20^ demonstrated this using data set from 498 IMRT plans with an AI method called Poisson regression. Lin et al,^21^ also developed a long short-term memory (LSTM) model using more than 1700 respiratory motions data from 3 institutions to predict various patient respiratory movements in real-time.

### Unsupervised learning

Unsupervised learning refers to the application of algorithms to derive conclusions from data sets that do not include labeled response in the form of input data. Cluster analysis is the most popular unsupervised learning approach because it is utilized for exploratory data analysis to uncover previously unseen patterns or groupings.^19^ The breathing curves of patients were analyzed by Li et al^22^ using the unsupervised learning methods of K-means and hierarchical clustering algorithms. These scientists separated patients into categories based on their breathing patterns, such as perfect, regular, and irregular. Using 341 real-time position management (RPM) files, they were able to extract the respiration signals and frequency spectrum.^22^

### Semi-supervised learning

Between supervised and unsupervised learning is semi-supervised learning. Some of the training data in semi-supervised learning lack labels. Yet, a model that describes the label part is created by taking use of the correlation structure of the input variables.^11^ Data from eight Linacs and seven institutions were used in a multi-institutional research by Naqa et al.^23^ The application of machine learning techniques for the automation of machine quality assurance was examined by the authors. The support vector data description clustering technique which is an example of a semi-supervised learning was fed with a total of 119 EPID (electronic portal imaging device) images of a unique QA phantom. Data from the multileaf collimator (MLC) offset, radiation field shift, and gantry sag were all included in the prediction tests. This research showed that support vector data description clustering and machine learning techniques are viable for creating automated quality assurance solutions.^19^

### Reinforcement learning

Reinforcement learning is a machine learning (ML) extension of traditional decision-making algorithms, Markov decision processes (MDPs). It focuses on how algorithms can interact with a given environment and take action.^15^ Typically, the algorithm must attain a specific objective by optimizing a cumulative reward function, such as therapeutic index in RT. The renowned Google AlphaGO is an example of application in which an algorithm learns how to win a board game under various circumstances.^19^ In RT, this can be applied to adaptive treatment planning, such as optimizing prescriptions for patients based on information gathered during treatment. In this instance, the algorithm will learn how to adapt dose fractionation based on the current condition of the patient undergoing RT (environment) in order to accomplish the objective of improved treatment response.^11,14,24^

### The influence of AI on the role of MPs in Radiotherapy Practice

#### Simulation

Simulation is a vital step in the RT treatment process, as it helps in preventing deficiencies or errors that can affect the treatment. Medical physicists mostly work together with radiation therapists and radiation oncologists. This process involves the use of various tools and techniques to simulate the various phases of the treatment, including registration, acquisition, and segmentation of images. Simulation typically involves using either MRI, CT imaging, or conventional simulator (diagnostic X-ray equipment with fluoroscopic capabilities). CT imaging has been the most widely used technique due to its high resolution, low cost-effectiveness, and availability. Although CT scans are commonly used in most centers, they can be enhanced with the help of MRI data through image fusion methods.^25,26^ Ideally, the fusion of images should be chosen to provide the best possible match for a specific RT task. In cases where CT images may not be accessible, it is possible to generate CT data from MRI without repetition of another exam. This helps save cost as well as dose reduction to the patient. This CT is called Synthetic CT (CTsynth) generated with AI methods. Images based on MRI data sets have been researched to highlight accuracy in tissues mapping.^8,27^ In a simulation, deep-learning neural networks which are also AI methods are now used by medical physicists to enhance the accuracy of RT simulation tasks by identifying objects of interest and reducing the effects of image artifacts. AI platforms are used by medical physicists to predict the radiation sensitivity of a tumor before treatment starts. This method helps determine the optimal dose to be given to the patient.^6^

Furthermore through functional image analysis, a radiobiological model can be created that can assist in the treatment of specific conditions. The medical physics community has greatly embraced computational modeling of radiobiological processes. This technology can be used for various purposes, such as the development of treatment plans and the optimization of protocols.^28^

#### Treatment planning

The RT planning process is quite complex. Due to the complexity, it can result in fatal mistakes or even cancer-causing radiation exposure. Medical physicists are also known to interact with the computer-assisted system to ensure that the plan is executed properly. RT treatment planning is an optimization problem that involves considerable freedom and is usually labor-intensive. The development of AI has led to new applications in this field, such as reducing human intervention and improving plan quality.^29^ AI studies are focused on the various steps of RT practice, such as the calculation of the dose, area determination, dose–volume histogram, and replanning in RT. The goal of these studies is to improve the accuracy of the RT planning process by identifying the most effective treatment technique for a given patient.^30,31^

AI-based treatment planning has been implemented in RT treatment planning by MPs, who use the data collected by trained algorithms to develop treatment plans that are based on detailed patient images and clinical data.^9,10^

Auto-planning which is as a result of this AI systems are also as good as their human-generated data plans. However, such plans would often have to be modified or customized by planning team members who are mostly medical physicists in many countries although some countries also have designated members called dosimetrists. The treatment planning process usually involves communicating with other team members to come up with a more effective and acceptable solution.^32^

#### Treatment delivery

In the course of RT treatment delivery, adjustments may be needed to ascertain that the treatment plan is being properly executed. The factors that can trigger the adjustment include the patient’s position and anatomical changes. Modern linear accelerators (Linacs) typically use cone beam CT (CBCT) for treatment confirmation. The use of CBCT has garnered wide acceptance due to its ability to visualize the position target in the patient. However, its low contrast images often provide images that are not as accurate as those from the planning scans. An AI-based algorithm has been developed and is used by medical physicists to enhance the resolution of CBCT images. This AI-based algorithm also provides a better and more accurate depiction of the target.^8^

However, imaging is not enough to identify soft tissue structures. The anatomical changes that occur during treatment can also warrant replanning. Some of these changes include the appearance of tumor shrinkage or a movement of the bowels, which could affect the doses being given to the organs. Replanning using AI is used by medical physicists to identify suitable patients for adaptive RT and to determine the real-time to execute this.

In relation to the various characteristics of a tumor, AI methods have been created and in use to see which patients will immensely derive gains most from modified treatment plans.^27^

#### Quality management systems

RT treatment delivery involves a lot of steps and measures are required to ensure the efficiency and effectiveness of each process. This would enhance the accuracy of doses delivered to patients, helping minimize toxicities associated with treatment delivery and improving treatment efficacy. Implementation of quality management systems in RT helps with the reposition of confidence in the treatment delivery. Quality management systems include quality assurance and control of equipment, radiation protection, and risk management:

##### Quality assurance

Quality assurance (QA) is a time-consuming and repetitive process that involves various tests to ensure that the treatment plan and the function of the linear accelerator meet specification. For many years, medical physicists have been implementing novel techniques and technology in clinical practice.^33^ QA is an important part of this process and plays a crucial task in the evaluation and reporting of the treatment plan.^26^ Following a treatment plan approved by the radiation oncologist, the medical physicist carries out a series of checks to make sure that all of its technical components meet acceptable guidelines.

The daily QA of RT is performed in cancer treatment to improve the quality of care. It is also used to enhance the understanding of the linear accelerator (linac) performance index and enable medical physicists to find out deviations in output. MPs are involved in validating and improving the dose predictions.^34^ AI-based methods have assisted MPs to carry out these roles faster and easier.^35^

In addition, MPs analyze and report on findings of AI-based tests related to linacs. They also assist in pointing out the cause of a matter and implementing corrective actions through rigorous testing of AI systems regularly.^32^ They are also expected to carry out a test program that has established goals and procedures.^24,36^

The frequency and nature of tests for AI systems need to be regularly updated to keep up with the latest developments in the field. Doing so helps keep the systems up to date with the latest learning and development techniques.^35^

##### Radiation protection and risk management

Many MP serves as radiation protection officers, they work in RT centers around the world to ensure the radiation protection of patients and staff. MPs are skilled to analyze and prevent accidents by through risk assessment. This process involves analyzing various events and scenarios to identify potential risks. Medical physicists use AI-based equipment to calibrate and readout doses from radiation protective devices such as dosimeters etc. AI can lower imaging radiation exposure, which can cause harm to patients and workers, without distorting the quality of the image.^37^ A recent study conducted by a medical physicist on using an AI-assisted chatbot for education on radiation safety and protection in RT revealed that using an AI-assisted chatbot helped the radiation staff members get the necessary knowledge about radiation safety. The chatbot’s character was built by machine learning to provide the necessary information to the radiation staff.^38^ Furthermore, a radiation protection data and device management platform called HERADO which uses AI and cloud-based technologies have been developed for automatic readout and transmission of radiation protection data.^39^ (Table 1)

**Table 1.** shows a summary of some selected publications with some examples of the medical physicists use of AI in radiotherapy practice

Authors/year of publication	Title of paper	AI method used	AI example in paper
Wang et al, (2020)	A predictive model of radiation-related fibrosis based on the radiomic features of magnetic resonance imaging and computed tomography	XGBoost	AI was used to predict radiation-related fibrosis of neck muscles based on MRI data from patients with nasopharyngeal carcinoma^40^
Lin. et al, (2019)	A Super-Learner Model for Tumor Motion Prediction and Management in Radiation Therapy: Development and Feasibility Evaluation	XGBoost	AI was used to predict tumour motion ranges using 4D CT images in patients receiving radiotherapy for lung cancer^41^
Mahdavi, et al, (2019)	Use of artificial neural network for pretreatment verification of intensity modulation radiation therapy fields.	Neural networks	In this study, AI was used to verify the pre-treatment doses of the 2D-fluence maps generated by an EPID in 60 patients receiving radiotherapy for prostate cancer and nasopharyngeal carcinoma.^42^
Xin et al, (2017)	Deep convolutional neural network with transfer learning for rectum toxicity prediction in cervical cancer radiotherapy: a feasibility study.	CNNs	AI was used to predict rectal toxicities of radiotherapy for cervical cancer.^43^
Tomori et al, (2018)	A deep learning-based prediction model for γ evaluation in patient-specific quality assurance.	CNNs	QA of dose distribution in patients receiving radiotherapy for prostate cancer.^44^
Marschner et al, (2022)	A deep image-to-image network organ segmentation algorithm for radiation treatment planning: principles and evaluation.	FCNN	AI was used for organ-at-risk segmentation in CT images of patients receiving radiotherapy to the thorax and pelvis regions.^45^
Maspero et al, (2018)	Dose evaluation of fast synthetic-CT generation using a generative adversarial network for general pelvis MR-only radiotherapy.	GANs	Generation of synthetic CT images using only MRI data to enable accurate calculation of radiation dose in the pelvis for patients with prostate, rectal and cervical cancer.^46^

AI, artificial intelligence; CNN, convolutional neural network; FCNN, Fully convolutional neural network; GAN, generative adversarial network.

### Education and training

Many medical physicist serves as heads of department in many educational institutions such as in universities and other research institutions. They provide education and training to many staffs who form part of the RT management team and to the general public. Some of these professionals include radiographers, oncology nurses, resident doctors, specialists. As the use of AI has increased, medical physics associations are developing educational programs that will help their members avoid making errors and minimize the possible risks associated with using AI devices.^7,34^ As medical physicists are skilled in communicating with patients and other healthcare professionals, they can also contribute to the development of AI modules in the education of medical physicist interns and students. They have also played key roles in the education and training in the application of AI for medical physics students and interns. The medical physics educational curriculum is also being modified in many countries to include a module on AI.^47,48^

### Research in AI

Aside from being active researchers and clinical scientists, MPs also have expertise in various other fields such as statistics and mathematics. To effectively introduce AI in clinical practice, the research should involve developing a comprehensive understanding of the characteristics of AI and performing data cleansing and validation.^49^ Medical physicists should also promote the use of digital information for the analysis of large data sets. This should involve the integration of various data sets in various areas. For AI research, the task of the MP is to define the problem that the AI system is trying to solve, and then select the appropriate models for testing and implementation. To improve the models, they need to be validated by well-defined steps.^5^

Due to the increasing importance of data collected in scientific and academic research, privacy, security, and access to information have become more critical. As a result, MPs should carefully consider the limitations of the data they're consuming when it comes to developing AI models.^35^ This is why the role of the MP has been assigned to oversee the development of policies and procedures related to these areas.

### Limitation MPs face with the use of AI in RT practice

Some of the major challenges MPs face with the use of AI include full interpretation. Interpretability refers to the level of understanding that a model has of the information it draws from data. This level of interpretability is very complex. Due to the complexity of the models involved in generating and analyzing the data, most AI models are typically perceived as black boxes. This issue is especially problematic for deep neural networks, which have many layers and multiple numerical operations involved in their development.^5^ Secondly, another limitation has to do with ethical issues associated with the automatic analysis of large patient databases including the privacy and data protection of individuals, the ownership of the data, and the quality of the training and validation of the models.^25^ The use of AI-based machines for tasks and decisions could introduce systemic risks. These risks are categorized into two categories: omissions and errors. The human responsibility to prevent these issues then falls on the shoulders of medical physicists. Thirdly, AI algorithms need a considerable large amount of training samples, which increases the data sizes medical physicists work with. Due to the complexity of the data sets, many AI algorithms require larger training samples. The lack of a proper metric to evaluate the power and size of the training samples can affect the prediction accuracy of AI models.^5,12^

## Conclusion

Medical physicists typically spend a lot of time providing advice and clinical services in RT centers. AI has the potential to reduce this time and make it possible to work seamlessly to improve quality care for cancer patients receiving RT.^50^ Despite the limitations with the use of AI in RT practice and its influence on the role of medical physicists has been thought of as a potential game changer in the precision and accuracy of treatment of cancer patients with RT.
